# The Heterochromatin Landscape in Migrating Cells and the Importance of H3K27me3 for Associated Transcriptome Alterations

**DOI:** 10.3390/cells7110205

**Published:** 2018-11-09

**Authors:** Tamar Segal, Mali Salmon-Divon, Gabi Gerlitz

**Affiliations:** Department of Molecular Biology, Faculty of Life Sciences and Ariel Center for Applied Cancer Research, Ariel University, Ariel 40700, Israel; tumer260@gmail.com

**Keywords:** chromatin, cell migration, histone modifications, transcription

## Abstract

H3K9me3, H3K27me3, and H4K20me1 are epigenetic markers associated with chromatin condensation and transcriptional repression. Previously, we found that migration of melanoma cells is associated with and dependent on global chromatin condensation that includes a global increase in these markers. Taken together with more recent reports by others suggests it is a general signature of migrating cells. Here, to learn about the function of these markers in migrating cells, we mapped them by ChIP-seq analysis. This analysis revealed that induction of migration leads to expansion of these markers along the genome and to an increased overlapping between them. Significantly, induction of migration led to a higher increase in H3K9me3 and H4K20me1 signals at repetitive elements than at protein-coding genes, while an opposite pattern was found for H3K27me3. Transcriptome analysis revealed 182 altered genes following induction of migration, of which 33% are dependent on H3K27me3 for these changes. H3K27me3 was also required to prevent changes in the expression of 501 other genes upon induction of migration. Taken together, our results suggest that heterochromatinization in migrating cells is global and not restricted to specific genomic loci and that H3K27me3 is a key component in executing a migration-specific transcriptional plan.

## 1. Introduction

Cell migration is one of cancer hallmarks that is fundamental for metastasis formation [1]. Tumor cell migration is highly dependent on cytoplasmic changes in the cytoskeleton and in the activity of motor proteins [2,3,4], but more recently alterations in chromatin structures were also found to be required [5]. Chromatin basic repetitive packaging unit is the nucleosome, which is composed of 147bp of DNA that are wrapped around an octamer of histones [6]. Nucleosomes are organized in higher order structures, of which relatively decondensed and transcribed regions are termed euchromatin, while more condensed and nontranscribed regions are termed heterochromatin. Euchromatin and heterochromatin are decorated by different levels and/or types of epigenetic marks, such as histone modifications. Heterochromatin-associated histone modifications include H3K9me3, H3K27me3, and H4K20me1 [7]. H3K9me3 is enriched in constitutive heterochromatin regions, such as repetitive elements and pericentromeric regions, where it is important for their repression [8]. Still, H3K9me3 was also found at promoters of repressed genes, as well as at some active genes [9]. H3K27me3 accumulates over cell-type specific repressed genes (facultative heterochromatin) and the inactivate X chromosome [9,10,11]. H4K20me1 is associated with repression of the inactive X chromosome and specific genes, but it was also found to accumulate downstream from the transcription start site (TSS) of highly active genes [12,13]. The inconsistent observations regarding H4K20me1 may arise from various cross-talks of H4K20me1 with other epigenetic marks and the fact that H4K20me1 is an initial step in the process of generating the highly repressive mark H4K20me3.

Previously, we found that migration of mouse melanoma cells is associated with and dependent on global chromatin condensation that includes more than a two-fold increase in the levels of H3K9me3, H3K27me3, and H4K20me1 [14,15,16]. More recently, migration-associated global chromatin condensation was reported in primary and transformed T-cells [17], primary tenocytes [18], and mesenchymal stem cells [19]. Reliance of cell migration on chromatin condensation has been reported in various types of cells, including lung cancer cells [20], embryonic fibroblasts [21], breast adenocarcinoma cells [22,23,24], colorectal cancer cells [24], prostate cancer cells [25], glioma cells [26], chondrosarcoma cells [27], epidermal cancer stem cells [28], primary tenocytes [18], and primary and transformed T-cells [17]. Thus, the dependence of tumor cell migration on chromatin condensation is a ubiquitous mechanism. Still, the exact roles of heterochromatin formation in migrating cells are not fully understood.

Here, to gain a mechanistic insight on the roles of heterochromatin in migrating cells, we mapped the changes in heterochromatin spread and in the transcriptome in melanoma cells in response to migration signals by ChIP-seq and RNA-seq, respectively. Induction of migration was associated with a more diffuse distribution of H3K9me3, H3K27me3, and H4K20me1 that generated lower number of peaks, but had a higher overlapping between the different histone modifications. Following induction of migration, H3K9me3 and H4K20me1 accumulated to a higher degree in repetitive regions, while H3K27me3 redistributed towards genes. In parallel, we identified 182 genes with altered RNA levels, of which one third were dependent on migration-induced methylation of H3K27.

## 2. Materials and Methods

### 2.1. Cell Culture

Mouse melanoma B16-F1 cells were bought from the ATCC and grown in DMEM (D5796, Sigma-Aldrich, St. Louis, MO, USA) supplemented with 10% FCS (04-007-1A Biological Industries, Beit Haemek, Israel), 0.292 mg/ mL L-glutamine (03-020-1B, 1A Biological Industries, Beit Haemek, Israel), and 40 units/mL Penicillin-Streptomycin (03-031-1B, Biological Industries, Beit Haemek, Israel) at 37 °C, 7% CO_2_. For migration assays, the cells plated on fibronectin-coated plates were grown to confluence. To induce migration, the cells were scratched at multiple sites, washed once with DMEM, and incubated in a growth medium at 37 °C and 7% CO_2_ for 3 h. Control cells were kept in similar conditions without being scratched. To inhibit the generation of H3K27me3 or to inhibit RNA polymerase II, the cells were incubated in a growth medium supplemented with 3 µM GSK343 (SML0766, Sigma-Aldrich, Rehovot, Israel) or 0.1 mM DRB (D1916, Sigma-Aldrich, Rehovot, Israel). The inhibitors were added 3 h before lysing the cells.

### 2.2. ChIP-seq

Chromatin immunoprecipitation (ChIP) was performed as described previously [29] with the following modifications. Following cross linking with 1% PFA, cells were collected and aliquoted to 10^7^ cells. Nuclei were isolated by lysis buffer and DNA was collected following fragmentation by 0.7 mU/μL of MNase (N3755, Sigma-Aldrich, Rehovot, Israel) at 37 °C for 15 min that was followed by a brief sonication in RIPA buffer. IP was done using magnetic protein A/G beads (BioVision, 6527-1, Milpitas, CA, USA) coupled to 24 µL of rabbit anti-H3K9me3 (Millipore, 07-442, Temecula, CA, USA), 12 µL of rabbit anti-H3K27me3 (Millipore, 07-449, Temecula, CA, USA), or 24 µL of rabbit anti-H4K20me1 (Millipore, 17-610, Temecula, CA, USA). DNA was purified using the QIAquick Gel Extraction kit (QIAGEN, 28704, Hilden, Germany) according to the manufacturer protocol and sequenced at the Technion Genome Center by an Illumina HiSeq 2500 machine.

### 2.3. RNA Purification and RNA-seq

Total RNA was purified by the NucleoZOL kit (MACHEREY-NAGEL, 740404.200, Duren, Germany) according to the manufacturer’s instructions. Purified RNA was sent for poly A containing mRNA selection, library preparation, and sequencing at the Technion Genome Center. Replicates number was five for untreated control and migrating cells and three for cells treated with GSK343 or DRB.

### 2.4. Peak Calling and Peak Analysis

Quality control checks of the raw sequence data were carried out using the FastQC tool (version 0.10.1) [30]. Then, the Trim_galore (version 0.4.1) [31] tool that is based on cutadapt [32] was used for adapters trimming and for removing of low quality bases from the ends of reads. The first four nucleotides, which were of bad quality, were trimmed from reads by a fastX trimmer (FATSX-Toolkit version 0.0.13) [33]. Cleaned, high-quality reads were aligned to the mouse genome (build mm10) using Bowtie2 [34] with the default parameters. PCR bias of duplicated reads were removed using the “MarkDuplicates” command implemented by the Picard tool (version 1.77) [35]. Broad peaks enriched in immunoprecipitation over input were identified by SICER [36] with “fragment size” of 350, “ effective genome fraction” of 0.8, and “ FDR” of 0.05. SICER-df was applied to determine differential peaks enriched in migrating cells compared to control cells and vice versa. Operations on genomic intervals were performed using BEDTools [37].

### 2.5. Calculation of ChIP-seq Coverage and Signal Distribution Across Specific Genomic Location

The genomic locations of Refseq genes and repetitive elements were downloaded from the UCSC table browser [38]. The enhancers’ locations were downloaded from HOMER [39] and the genomic coordinates were converted to mm10 assembly using the UCSC liftOver tool. Promoters were defined as 1000 bp upstream of transcription start sites (TSS). Within each list, overlapping intervals were merged into a single feature that spans all of the combined features. In order to avoid intervals overlapping more than a single defined annotated element, we removed from each list nucleotides that belong to another annotation list by assigning the following priority: Coding genes, noncoding genes, promoters, enhancers and repetitive elements. The coverage of the ChIP signal across these regions was presented as a percentage of reads mapping within genomic regions out of the total mapped reads without redundancy, or as a percentage of bp mapping within genomic regions out of the total number of bp included within differential peaks, and was calculated using coverageBed [37] and intersectBed [37], respectively. The distribution of ChIP signal across functional genomic regions was done using ngs.plot [40].

### 2.6. Correlation Analysis and Combinatorial Pattern of Heterochromatin Markers

The entire genome was divided into non-overlapping equally sized bins (10 kb), and the average ChIP score was calculated for each bin using the multiBamSummary command from deepTools [41]. Spearman correlation coefficients were computed using deepTool’s multiBamSummary and plotCorrelation commands. Hidden Markov model-based chromatin state definition was performed using the ChromHMM v1.14 [42]. Five states were used to segment the genome, and the enrichment of these states across different genomic features was calculated.

### 2.7. RNA-seq Analysis

Reads were aligned to the mouse genome (mm10) using TopHat [43] after removal of adapter sequences and critical examination of quality controls using Trim_galore [31] with the default parameters. The number of reads mapping to each mouse gene (as annotated in Ensembl release GRCm38.86) was counted using the ‘intersection-nonempty’ mode of HTseq-count script [44]. Differential expression analysis was performed using the edgeR [45] and Limma [46] packages from the Bioconductor framework [47]. Briefly, features with less than 1 read per million in 3 samples were removed. The remaining gene counts were normalized using the TMM method, followed by voom transformation [48]. Linear models were used to remove the batch effect and to find differentially expressed genes. Up- and down-regulated genes having FDR <0.05 and fold change ≥1.3 were analyzed for pathway enrichment using the Ingenuity Pathway Analysis [49] (IPA). H3K27me3 dependency was detected by comparing genes found to be differentially expressed between migrating and control cells to genes found to be differentially expressed between migrating cells treated with GSK343 to control cells. H3K27me3-dependent genes are genes whose expression changes abolished upon treatment with GSK343, while H3K27me3-independent genes are those whose change in expression was preserved even after treatment with GSK343.

### 2.8. Distribution of Heterochromatin Signals Across High and Low Abundant Genes

Average gene expression was calculated across five replicates of RNA-seq samples for control cells and migrating cells and genes with an average expression which was lower than 1 cpm (counts per million) reads were excluded. Genes were ranked based on their expression level, and the top and bottom 500 genes were chosen. The distribution of histone modifications signal across these genes was plotted using the ngs.plot tool [40].

## 3. Results

### 3.1. Migration-Induced Changes in the Genomic Distribution of H3K9me3, H3K27me3, and H4K20me1

Induction of migration leads to a global increase of 2–4-fold in the levels of the heterochromatin markers H3K9me3, H3K27me3, and H4K20me1, as we previously detected by immunostaining [14,15,16]. To learn about the mechanistic role of these histone modifications in the migration process, we mapped their relative distribution along the genome by ChIP-seq. Migration of the B16-F1 mouse melanoma cells was induced by the wound healing assay [50], in which multiple scratches were performed in each plate to generate large numbers of migrating cells. This way of induction of migration enabled us to receive an enriched population of migrating cells as measured by a 2–5-fold increase in the levels of H3K9me3 and H3K27me3 at the promoters of *E-cadherin*, *Gapdh*, and *Line* (Figure S1).

**Figure 1:**
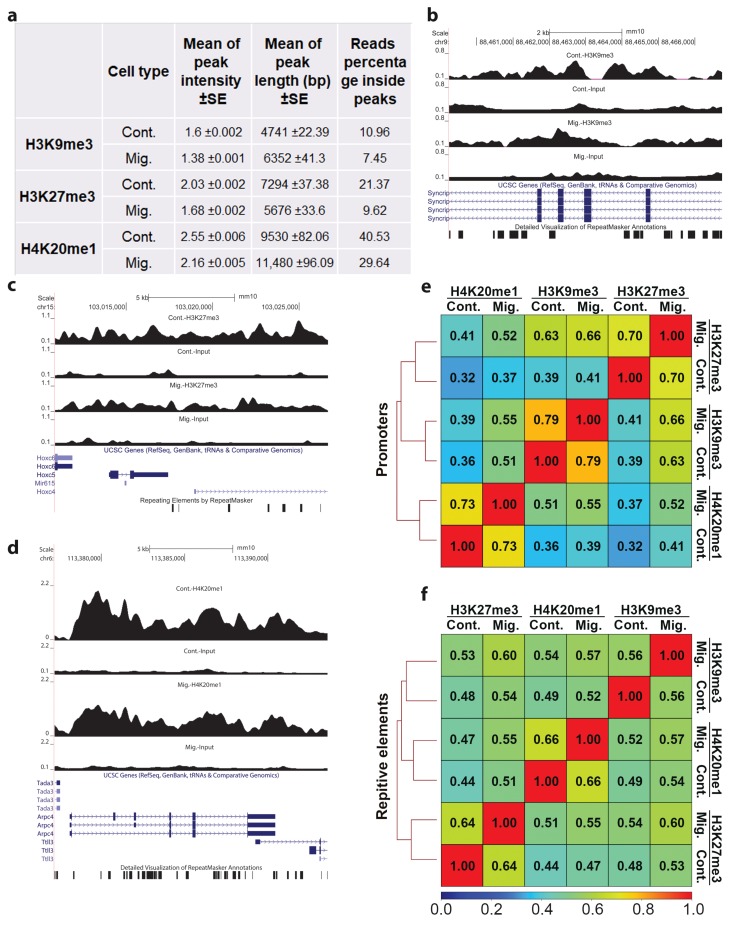
The patterns of the ChIP-seq signals of H3K9me3, H3K27me3, and H4K20me1 upon induction of migration. (**a**) Mean ± SE of ChIP-seq peak intensities and lengths of H3K9me3, H3K27me3, and H4K20me1 in control (Cont.) and migrating (Mig.) cells. Reads percentage inside peaks are the percentages of ChIP-seq mapped reads of the indicated heterochromatin markers that are localized inside peaks. Statistical significance was calculated between control cells to migrating cells by Wilcoxon rank sum test, *p* < 2.2 × 10^−16^. (**b**–**d**) UCSC browser shots of H3K9me3, H3K27me3, and H4K20me1, respectively. (**e**,**f**) Correlation between the three heterochromatin markers over promoters and repetitive elements. Spearman correlation coefficients of the ChIP-seq signals were calculated from reads coverage of consecutively equally sized 10 kb bins.

H3K9me3, H3K27me3, and H4K20me1 ChIP-seq-mapped reads were at the range of 27–42 million and the coverage values at the range of 0.68–1.52 (Figure S2a). As expected from the nature of heterochromatin modifications, more than 50% of the signals of these modifications did not accumulate at defined and short loci to form sharp peaks (Figure 1a). Moreover, following indication of migration, this phenomenon further increased, resulting in the accumulation of only 7.45%, 9.62%, and 29.64% of the reads of H3K9me3, H3K27me3, and H4K20me1, respectively, inside peaks (Figure 1a). In agreement, upon induction of migration, the intensities of the peaks were reduced by 14–17% (Figure 1a–d), and the number of identified peaks was reduced by 30–40%, while the number of differential peaks was reduced by more than 90% (Figure S2b). Significantly, upon induction of migration, the average peak length of H3K9me3 and H4K20me1 was increased by 34% and 20%, respectively, while the average peak length of H3K27me3 was reduced by 20% (Figure 1a–d). Taken together, the above analyses indicate more diffused signals of H3K9me3, H3K27me3, and H4K20me1 upon induction of migration. This pattern suggests a possible increase in the degree of overlap between the three modifications following induction of migration. Indeed, in migrating cells, the correlation between these modifications increased significantly over any evaluated genomic element (promoters, repetitive elements, enhancers, and gene bodies) (Figure 1e,f and Figure S3).

**Figure 2:**
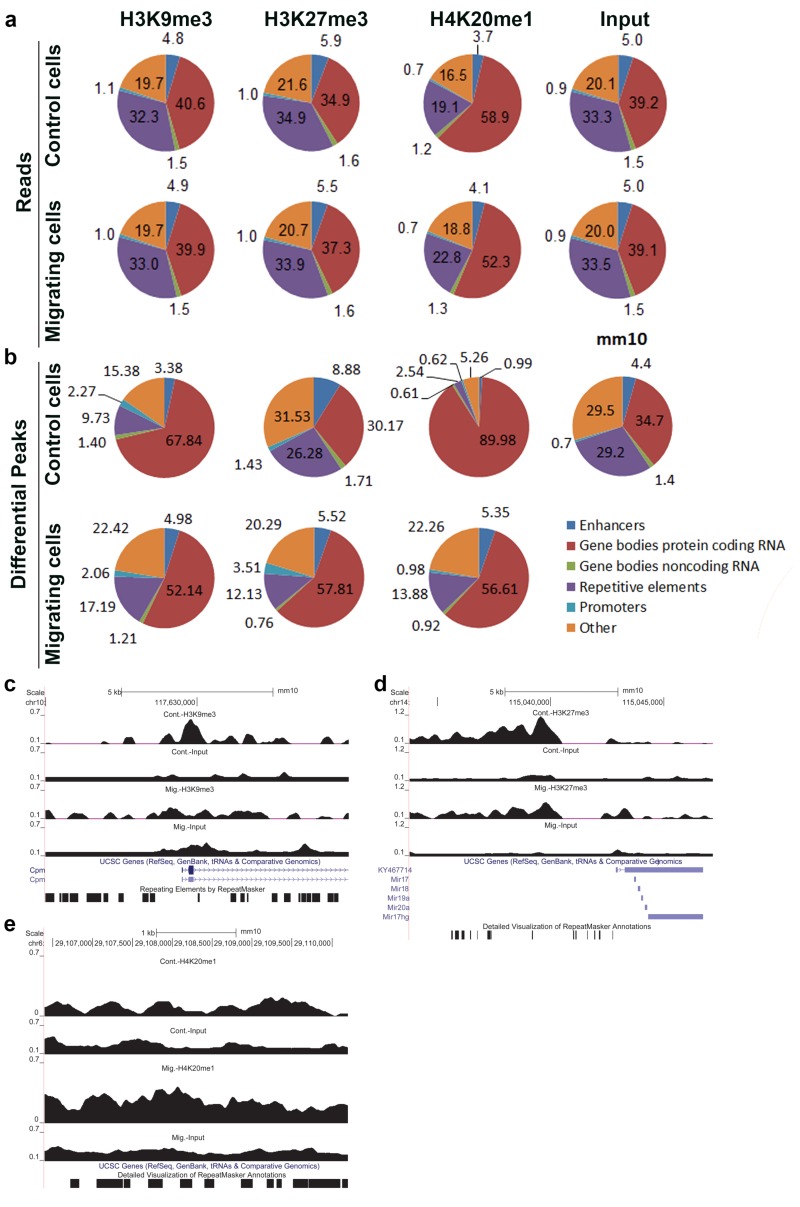
Relative distribution of ChIP-seq signal across various genomic elements in control and migrating cells. (**a**,**b**) The relative distribution of ChIP-seq reads (**a**) and ChIP-seq differential peaks (**b**) across the indicated genomic elements was calculated for the input and the three heterochromatin markers in control cells and in migrating cells. (**c**–**e**) UCSC browser shots of H3K9me3, H3K27me3, and H4K20me1, respectively.

To assess which genomic regions are more prone to being affected in migrating cells by each of the above modifications, we counted the number of mapped reads overlapping specific genomic regions and calculated them as the percentage of the total mapped reads (Figure 2a). We also calculated the relative distribution of differential peaks that fall inside different genomic elements (Figure 2b).

This analysis revealed a migration-induced increase in the relative distribution of nucleotides in differential peaks of H3K9me3 and H4K20me1 at repetitive elements by 83% and 446%, respectively, and a migration-induced decrease of these modifications at protein-coding genes by 23% and 37%, respectively. On contrary, upon induction of migration, the relative distribution of nucleotides in differential peaks of H3K27me3 increased by 92% at protein-coding genes, while it decreased by 54% at repetitive elements (Figure 2b). A similar trend was seen in the relative distribution of the total reads of these modifications, as well (Figure 2a,c–e).

**Figure 3:**
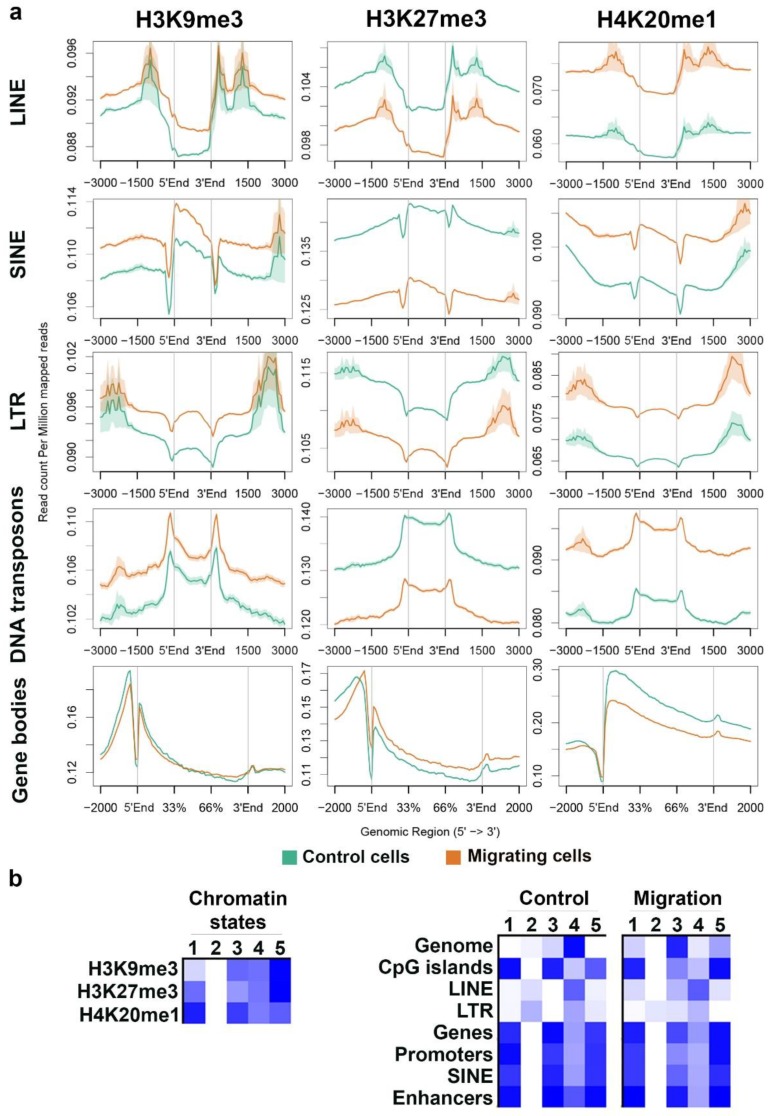
Distinct and combinatorial pattern of heterochromatin markers over genomic elements. (**a**) Distribution of H3K9me3, H3K27me3, and H4K20me1 ChIP-seq signals in control cells (green line) and in migrating cells (orange line) across gene bodies and the repetitive elements LINE (n = 671,156), SINE (n = 682,126), LTR (n = 646,284), and DNA transposons (n = 79,756). In each graph, the region between 5’End and 3’End represents the analyzed element. (**b**) Five combinatorial chromatin states were defined using the three heterochromatin markers. The left panel describes the chromatin states as represented by the emission coefficients in ChromHMM model; the right panel describes the relative enrichment of states coverage across the whole genome and over different genomic regions, in control and migrating cells.

To verify these results, we analyzed the average signal distribution of these modifications across different types of repetitive elements and across protein-coding genes. In agreement with the previous analysis, the signals of H3K9me3 and H4K20me1 were higher across LINE, SINE, LTR, and DNA transposons in migrating cells than in control cells, while the signal of H3K27me3 was lower across the same repetitive elements in migrating cells than in control cells. An opposite pattern emerged over protein-coding genes: Reduced levels of H4K20me1 together with increasing levels of H3K27me3 in migrating cells than in control cells (Figure 3a).

Classifying the combinatorial pattern of the above modifications using a hidden-Markov-model (HMM) based approach revealed that upon migration, there is a reduction in the genomic coverage by regions with similar levels of the three markers (state no. 4, Figure 3b), while there is an increase in the percentage of genomic regions that are decorated by all three markers, but at differential levels (states no. 1, 3, and 5, Figure 3b). Interestingly, regions free of all three markers occur in repetitive elements (LINE and LTR), but their percentage decreases upon induction of migration (state no. 2, Figure 3b).

Importantly, upon induction of migration, the changes in the combinations of the modifications across promoters and gene bodies were much smaller than the changes over the whole genome, thus raising the question if there are any transcriptional changes in migrating cells.

### 3.2. Migration-Induced Transcriptome Changes

**Figure 4:**
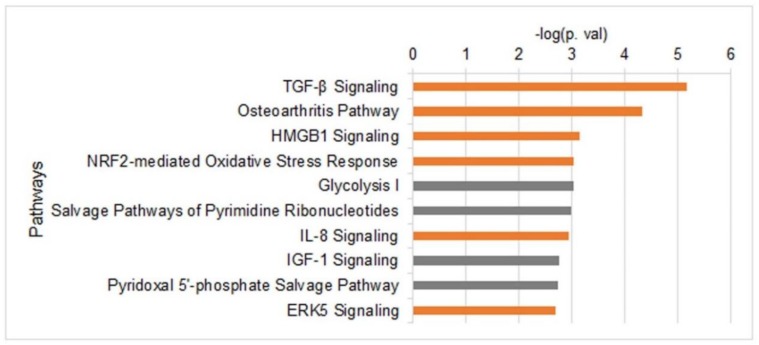
Altered gene expression upon induction of migration. Top 10 pathways that are altered upon induction of migration as were identified by IPA using RNA-seq data collected from control cells and migrating cells (n = 5). Only genes that were differentially expressed by a factor of >1.3 and FDR <0.05 were included in the analysis. Orange bars represent up-regulated pathways and grey bars represent altered pathways in which the altered genes did not have a uniform trend.

To determine the changes in the transcriptome upon induction of migration, we carried out an RNA-seq analysis (Figure S4). Differential expression analyses identified 801 genes with altered expression levels (FDR < 0.05) in cells that were induced to migrate for 3 h; 465 genes were up-regulated and 336 genes were down-regulated. Out of them, 182 genes were altered with a fold change of >1.3; 129 up-regulated genes and 53 down-regulated genes (Table S1 and Figure S5). Surprisingly, although induction of migration induces a global increase in chromatin condensation, a higher number of genes were up-regulated than down-regulated. To search for common functions of the altered genes, genes found to be differentially expressed with FDR >0.05 and fold change >1.3 were subjected to Ingenuity Pathway Analysis (IPA). As shown in Figure 4, the most significantly enriched pathways included ones that are known to be associated with tumor cell proliferation and migration, such as the TGF-β, IGF-1 and ERK5 signaling pathways [51,52,53,54,55], and pathways involved in energy generation: Glycolysis and pyridoxal 5′-phosphate (PLP) salvage pathways. PLP is an active form of vitamin B6, which is important for the metabolism of carbohydrates, amino acids, and fats, the generation of the methyl donor S-adenosylmethionine (SAM), and in neutralizing oxidative stress [56,57]. Activation of oxidative stress response was also indicated by the up-regulation of NRF2 targets (Figure 4). Analysis of upstream regulators and downstream-affected functions identified a strong link to cell migration (Figure S6).

To evaluate the correlations between H3K9me3, H3K27me3, and H4K20me1 and transcription in our system, we assessed the spread of each modification over the genes that are found in peak areas of the same modification. H3K9me3 enrichment was not found at unexpressed genes, which may be repressed by DNA methylation. In expressed genes, H3K9me3 enrichment along gene bodies correlated with higher gene expression levels, while H3K9me3 enrichment at promoters correlated with lower gene expression levels (Figure S7: Cluster no. 1 versus cluster no. 3, respectively). A similar pattern was found for H4K20me1: High enrichment levels of H4K20me1 along gene bodies correlated with high gene expression levels, whereas high enrichment levels of H4K20me1 at promoters or its moderate enrichment over gene bodies associated with low gene expression levels (Figure S8). Significantly, the correlation of H3K27me3 enrichment with low gene expression was the most pronounced out of the three evaluated modifications (Figure S9).

### 3.3. H3K27me-Dependent Transcriptome Changes upon Induction of Migration

**Figure 5:**
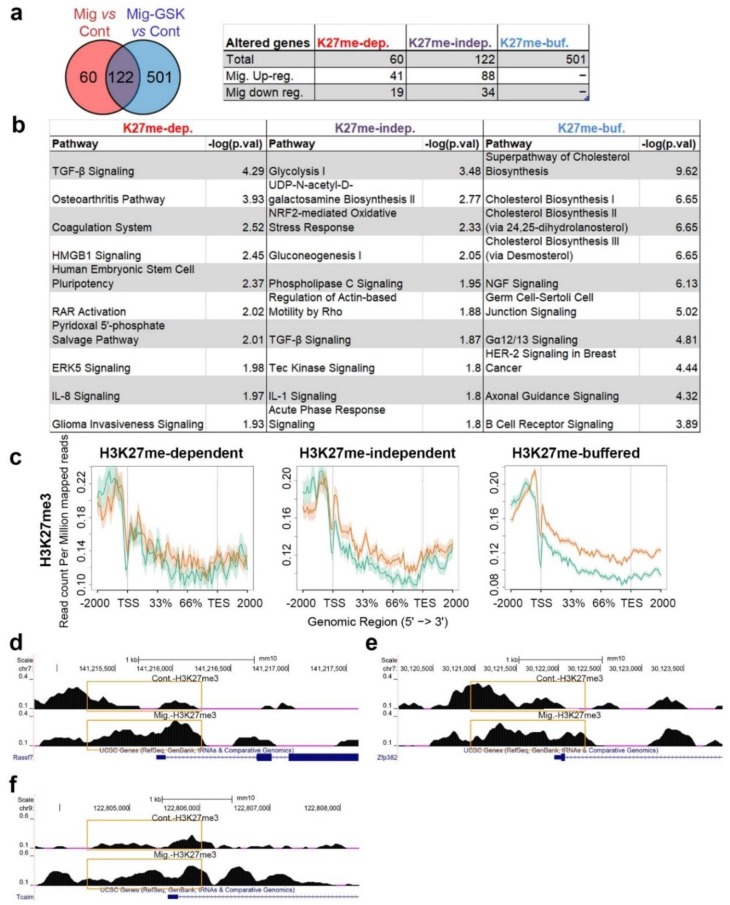
The dependence of migration-induced transcriptional changes on H3K27 methylation. (**a**) Altered genes upon induction of migration (fold change > 1.3, FDR < 0.05) that were detected in untreated migrating cells and in migrating cells treated with GSK343 are represented by a Venn diagram. Overlapping genes are termed “H3K27me-independent”, genes altered only when migrating cells were compared to control cells are termed “H3K27me-dependent”, and differentially expressed genes only in GSK343-treated migrating cells are termed “H3K27me-buffered”. (**b**) Top 10 pathways altered according to the “H3K27me-independent” gene list, the “H3K27me-dependent” gene list, and the “H3K27me-buffered” gene list as identified by IPA. (**c**) Distribution of H3K27me3 signal across “H3K27me-dependent”, “H3K27me-independent” and “H3K27me-buffered” genes in control cells (green) and in migrating cells (orange). The region between 5’End and the 3’End represents genes bodies. Additional 2000 bp upstream and downstream of the genes are plotted. (**d**–**f**) H3K27me3 signal around the transcription start site (TSS) of *Rassf7*, *Zfp382*, and *Tcaim.* The altered signals around the TSS are marked by orange rectangles.

Out of the three analyzed histone modifications, only H3K27me3 displayed increased levels over genes upon induction of migration (Figure 3). Therefore, we analyzed which migration-associated transcriptome changes are dependent on H3K27me3 by RNA-seq of migrating cells that were treated with the Ezh2-specific inhibitor, GSK343, at a concentration that we previously found to inhibit migration [16]. This analysis revealed that 33% of the migration-altered genes were not changed once GSK343 was added, indicating they are H3K27me3-dependent genes. The 67% of the migration-altered genes that were changed also in migrating cells treated with GSK343 were termed H3K27me-independent genes (Figure 5a, Table S2, Figures S4 and S10). Significantly, most of the signaling pathways altered in migrating cells, such as TGF-β and ERK5, were H3K27me-dependent, while the most significant H3K27me independent-pathways were glycolysis and NRF2-mediated response (Figure 5b). In addition, 501 genes that normally do not change upon induction of migration did change once H3K27 methylation was inhibited (Figure 5, Table S2, Figure S10). These genes (termed H3K27me-buffered genes) are mainly involved in cholesterol metabolism. Thus, H3K27 methylation in migrating cells is important not only for establishing a new transcriptional plan, but also to limit the transcriptional changes to specific genes. Analyzing the levels of H3K27me3 at the promoters of H3K27me-buffered genes revealed a clear increase in H3K27me3 levels upon induction of migration that was not found in the H3K27me-indepednent genes (Figure 5c). A similar analysis of the H3K27me-dependent genes did not find significant changes between control and migrating cells, probably due to the low number of genes in this group.

Still, examination of single H3K27me-dependent down-regulated genes identified increased H3K27me3 levels around the TSS in most of them, including *Rassf7*, *Zfp382*, and *Tcaim* (Figure 5d–f). Significantly, these genes can affect the expression of additional genes since they are involved in signaling (*Rassf7* and *Tcaim*) or in transcriptional control (*Zfp382*).

### 3.4. H3K27me-Dependent Genes Are Less Prone to RNA Stability Control

**Figure 6:**
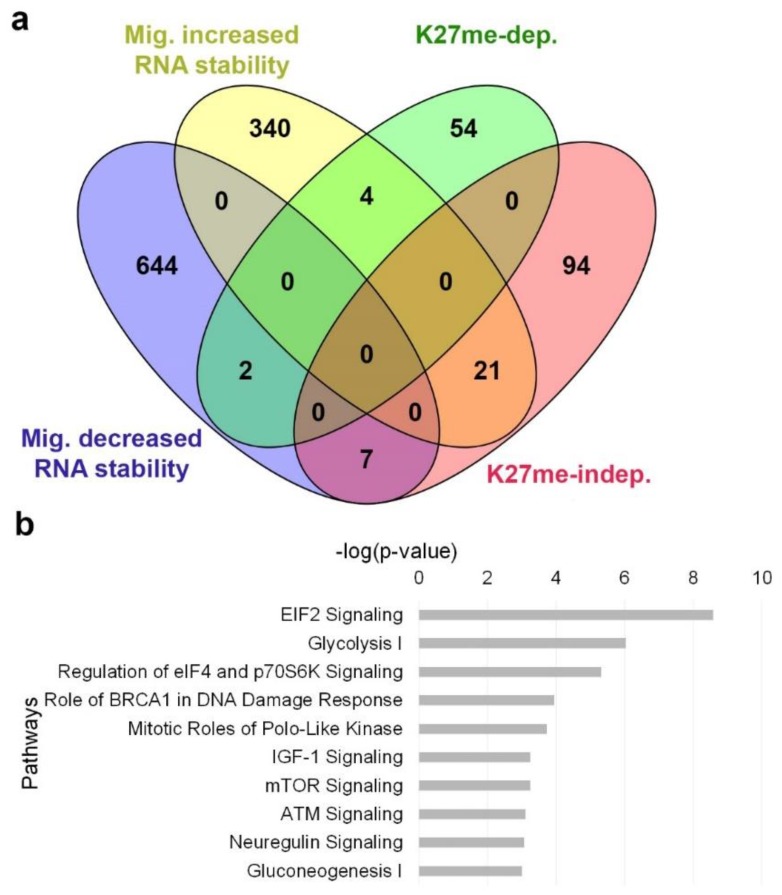
Migration-induced changes in RNA stability. Altered genes upon induction of migration were detected in the presence of DRB, an RNA polymerase II inhibitor (fold change > 1.3, FDR < 0.05). Increased RNA stability is detected by up-regulation of genes in DRB-treated migrating cells compared to DRB-treated control cells, and decreased RNA stability by down-regulation. (**a**) A Venn diagram representing the overlap between genes with increased or decreased RNA stability levels upon induction of migration to H3K27me-dependent and independent genes. (**b**) Top 10 pathways of the genes with altered RNA stability levels upon induction of migration as identified by IPA.

Finding that 67% of the migration-altered transcripts are independent of H3K27 methylation means that additional mechanisms are involved in their regulation. To learn if alterations in their RNA stability levels are involved, we determined the transcriptome of control and migrating cells following 3 h of RNA polymerase II inhibition by DRB. Calculation of the ratio between the mRNA levels in DRB-treated migrating cells and DRB-treated control cells identified 653 down-regulated genes and 365 up-regulated genes having decreased and increased RNA stability upon induction of migration, respectively (Figure 6a, Table S3, Figures S4 and S11). Notably, whereas only 10% of the H3K27me-dependent genes had migration-dependent changes in their RNA stability levels, 23% of the H3K27me-independent genes had migration-dependent changes in their RNA stability levels. IPA analysis revealed that genes with increased RNA stability in migrating cells are mainly involved in translational control and energy production, while genes with decreased RNA stability in migrating cells are mainly involved in cell cycle control and DNA damage response (Figure 6b), suggesting that regulation at the levels of RNA stability and translation rate may be highly important for cell migration.

## 4. Discussion

In this study, we found that upon induction of migration, the increase in heterochromatin markers does not occur in narrow regions to generate peaks, but rather spreads over larger genomic regions. Migration signals alter the RNA levels of 182 genes that are involved in signaling pathways, such as TGF-β and ERK5, but also in glucose metabolism and oxidative stress response. One third of the transcriptome alterations is dependent on methylation of H3K27, while altered genes in an H3K27me-indepednent manner are more prone to regulation at their RNA stability level.

ChIP-seq protocols depend on the assumption that the overall yields of DNA are identical per cell under different conditions. Accordingly, the same amounts of total DNA are taken for analysis and the resulting data are normalized to each other, so that the total amounts of signals are identical. Since induction of migration is associated with a dramatic increase in the amount of heterochromatin, ChIP doesn’t yield the same amount of DNA in control and migrating cells. Hence, the DNA normalization done during library preparation reduces the quantitative difference between the two conditions, which explains the lack of global increase in heterochromatin markers upon induction of migration in our ChIP-seq experiments. Therefore, our analysis has been mainly focused on the changes in signal distribution across genomic features upon induction of migration.

Induction of migration led to a more diffuse distribution of H3K9me3, H3K27me3, and H4K20me1 along the genome as measured by the reduced number of ChIP-seq peaks and ChIP-seq signals within peaks (Figure 1 and Figure S2). In agreement with this pattern, the overlapping between the different markers increased dramatically (Figure 1). These changes were accompanied by a shift in the distribution of these markers between the various genomic elements: H3K9me3 and H4K20me1 signals shifted from protein-coding regions to repetitive elements, while the H3K27me3 signal shifted in the opposite direction (Figure 2). These patterns suggest that although the overall signals of each of these markers is increased by 2–4-fold, as previously measured by immunostaining [14,15,16], the effects on transcription may be moderate, since the signals do not accumulate at narrow regions. Indeed, transcriptome analysis revealed that upon induction of migration, only 182 genes were altered with a fold change of >1.3, while only 30% of them were down-regulated.

Reports on the transcriptome of migrating cells are quite scarce [58,59,60]. In previous reports, the transcriptome was evaluated by microarrays that had a limited genomic coverage. Analyzing our RNA-seq data by IPA identified the activation of TGF-β IL-8 and ERK5 signaling pathways that were found to be activated in previous transcriptome analyses of migrating cells [58,59,60] (Figure 4). These pathways are well-established regulators of cancer cell migration and invasion [51,52,53,54,55]. In addition, we found an increase in three glycolytic enzymes (Glucose-6-Phosphate Isomerase, Phosphofructokinase, and Aldolase C) that can increase energy production in migrating cells. The increase in oxidative stress response genes may be required due to the increase in ATP production.

Analyzing the association of H3K9me3, H3K27me3, and H4K20me1 with gene expression levels revealed a positive correlation between high H4K20me1 levels downstream the TSS and gene expression levels, and negative correlation between H4K20me1 accumulation upstream the TSS and gene expression levels (Figure S8). Thus, H4K20me1 may support transcription when localized downstream the TSS, whereas it may be involved in transcriptional repression when localized upstream of the TSS or at low levels over the gene body. We also found a positive association between H3K9me3 enrichment along gene bodies and high expression levels (Figure S7), as found before [61,62].

Using an Ezh2 specific inhibitor, we were able to find that methylation of H3K27 is required for the transcriptional regulation of 33% of the migration-altered genes, as well as to prevent changes in 501 additional genes upon induction of migration (Figure 5). Migration signals lead to the activation of several transcriptional regulators, such as SMAD4 and SP1 (Figure S6) that have the potential to affect the transcription of several hundreds of genes. We hypothesize that the changes in the transcription of many of these genes are not required or may even interfere with the migration process, and H3K27me3 serves to prevent the binding or the activity of transcription factors at these genes. H3K27 methylation is mainly known for its negative effect on transcription; however, the group of H3K27me-depedent genes includes 41 up-regulated genes and 19 down-regulated genes. We hypothesize that only a fraction of these genes is affected directly by H3K27 methylation and this fraction of genes regulates the expression of many additional genes. In support of this hypothesis, we identified increased H3K27me3 levels at the promoter of many of the H3K27me-depedent migration-repressed genes, such as *Rassf7*, *Zfp382*, and *Tciam* (Figure 5d–f). Interestingly, Rassf7 was shown to inhibit the MKK7/JNK signaling pathway [63], Zfp382 was shown to inhibit the transcription of various tumor and migration promoting genes [64,65], and Tciam was found to inhibit LPS signaling and NF-κB activity [66].

In analyzing the importance of transcription for cell migration, we previously found that active transcription is dispensable for migration during a time period of 3 h, whereas it is important for migration during a longer period of time (11 h) [14]. Thus, the changes we identified here are the initial changes that will have an accumulative effect as the migration time increases. The spread of the heterochromatin markers upon induction of migration may indicate additional roles for these markers during cell migration such as increasing nuclear rigidity [5,18,67,68]. Overall, our results support a model in which migration-induced chromatin condensation serves to alter both the transcription plan as well as the physical properties of the nucleus.

## Data Availability

The raw data files of sequencing experiments have been deposited in the sequence read archive, accession number PRJNA437732.

## Supplementary Materials

Supplementary figures, tables, scripts and methods are available online at http://www.mdpi.com/2073-4409/7/11/205/s1.
