# Supplementary material for: The Heterochromatin Landscape in Migrating Cells and the Importance of H3K27me3 for Associated Transcriptome Alterations
Source: Cells. 2018 Nov 9;7(11):205. doi: 10.3390/cells7110205 (PMC6262444; doi:10.3390/cells7110205)
Supplement: Supplementary file 1 [file cells-07-00205-s001.zip › Supplementary_Materials.pdf]

The heterochromatin landscape in migrating cells and the importance of H3K27me3 for migration-associated transcriptional changes

Tamar Segal, Mali Salmon-Divon and Gabi Gerlitz

Supplementary Methods

ChIP-qPCR. The described ChIP-seq protocol was scaled down by a factor of 4, thus for each point lysate from $0.25*10^7$ was taken. In addition to the above antibodies an additional IP point was carried out with 3µl of control rabbit IgG (Millipore, PP64B, CA, USA). Isolated DNA subjected to qPCR using Fast SYBR® Green Master Mix (Applied Biosystems, 4385612, Vilnius, Lithuania), according to the manufacturer's protocol. Each experiment was repeated three times in duplicates. Standard errors (SE) and Wilcoxon signed-rank test were calculated. A list of the primers used appears in Sup. Table 1.

Reverse transcription of mRNA and qPCR. Validation of the RNA-seq results of selected mRNAs was performed by reverse transcription followed by qPCR. In short, mature RNA was reverse-transcribed by GoScript™ Reverse Transcription System using polyT primers according to the manufacturer's protocols (Promega, A5000, Madison, USA). cDNA was subjected to qPCR using Fast SYBR® Green Master Mix (Applied Biosystems, 4385612, Vilnius, Lithuania), according to the manufacturer's protocol. The relative expression was normalized to RPS29 and each experiment was repeated at least three times in duplicates. Standard errors (SE) and T.test were calculated. A list of the primers used appears in Sup. Table 1.

Heatmap of ChIP signal across genes. A matrix containing the normalized ChIP-seq score across ±3000 bp around the TSS of Refseq genes was prepared using bwtool [1]. To keep intervals in 5'→3' direction, regions around the TSS of genes transcribed from the negative

strand of the reference genome were reversed. An in-house script was written to calculate the average score across every 20 bp, leading to a signal matrix of 300 signal points around the TSS per gene. K mean clustering algorithm was applied to divide the genes into clusters based on signal intensity around their TSS. The "elbow" method [2] was used in order to determine the optimal number of clusters. Briefly, a plot of the "within groups sum of squared errors" by the "number of clusters extracted" was generated. The estimated optimal number of clusters is the number under which the elbow or bend in the plot is found. Heatmaps presenting the distribution of TSS signal within each cluster were created by Complex Heatmap [3] and EnrichedHeatmap [4] R packages. For correlation calculation, the maximum signal value across 300 signal points was detected for each gene, and Pearson correlation coefficient was computed between these modification signal value and the gene expression level. For H4K20me1, correlation calculation was done separately for signal upstream the TSS and downstream the TSS.

Primers used in PCR analysis

**Gene name**	**Forward primer (5'→3')**	**Reverse primer (5'→3')**
E-cadherin	ACTTCTGCTAGACTCCTGCC	ATCATGGGGCACGAGTCAG
Gapdh	TTGTGCAGTGCCAGGTGAAAA	CCATCACGTCCTCCATCATCC
Line	GCCTAAGCCACAGCAGCAG	ACACAGGGTGCTGCCTCAG
Ctgf	CCTGCGACCCACACAAGG	GCTGCTTTGGAAGGACTCAC
Cyr61	AGACCCGGATCTGTGAAGTG	GGGCCGGTATTTCTTGACAC
Fos	AGAGCGGGAATGGTGAAGAC	CTGCAGCCATCTTATTCCGT
Id1	CTGAACGGCGAGATCAGTG	TTTTCCTCTTGCCTCCTGAA
Mgarp	TGGAACATCTGGCTCCAATA	CAACTCCGCCTTTGTTTGTT

Nppb	GCACAAGATAGACCGGATCG	CTTCAAAGGTGGTCCCAGAG
Plk2	CAGATGCCCCTGAACAATTT	GGAGGTAGAGCCGAGGTCTT
Ddit4	ACCAGTTCGCTCACCCTTC	AAACGATCCCAGAGGCTAGG
Hk2	GGCCGTGGTAAATGACACAG	CCACCAGTTCCACATTACGC
Kdm3a	AAGGACCAGCAGAAGGACTC	GCCAATCGCTTCACTGTCAT
Tnfsf9	ACTACACAACAGGGCTCTCC	GTCTTCTTCGTACCTCAGACCT
Bmt2	CACTGGGCAAAAACTTGTGA	GTCTTAGTGGCGAGGACAGC
Narf	GAAGGGCAAACTCCCATACC	CCGGAGTAGATCCCTTCCAT
Slc2a1	TTGTTGTAGAGCGAGCTGGA	AAGGCCACAAAGCCAAAGAT
Rps29	TCGTTGGGCGTCTGAAGGCAA	CGGAAGCACTGGCGGCACAT
Serpinb9b	CCAGAGTTGTTGGCAGATGAC	ACTGGCCTTTTCTCATCCTTGT
Selenbp1	CTAACCCACAGAAGCCCCG	GCTGGATCATCTGAGGGCC
Runx2	TGAGATTTGTGGGCCGGAG	AGCTTCTGTCTGTGCCTTCT
Bmt2	CACTGGGCAAAAACTTGTGA	GTCTTAGTGGCGAGGACAGC
Tnfsf9	ACTACACAACAGGGCTCTCC	GTCTTCTTCGTACCTCAGACCT
Pgbd5	TACAAGGTCCAGCCCTTCCT	TTTCCGCTTTTTCCTCTTCC
Hsd17b7	GGACGTTGCTCCTACCCATA	CGCGTATTTGGTCAGAGGAT
Frs3	GTGTTCGAGGGCAGAGGAC	CAGAGACCGTGTAGCCAGC
Adamts1	GACCCGAGAGCCAGAACAC	CTACGAGCATGGTTTCCACA

References

[1] A. Pohl, M. Beato, bwtool: a tool for bigWig files, Bioinformatics. 30 (2014) 1618–1619. doi:10.1093/bioinformatics/btu056.

[2] D.J. Ketchen, C.L. Shook, The Application of Cluster Analysis in Strategic Management Research: An Analysis and Critique, Strateg. Manag. J. 17 (1996) 441–458. doi:10.1002/(SICI)1097-0266(199606)17:6<441::AID-SMJ819>3.0.CO;2-G.

[3] Z. Gu, R. Eils, M. Schlesner, Complex heatmaps reveal patterns and correlations in multidimensional genomic data, Bioinforma. Oxf. Engl. 32 (2016) 2847–2849. doi:10.1093/bioinformatics/btw313.

[4] Z. Gu, R. Eils, M. Schlesner, N. Ishaque, EnrichedHeatmap: an R/Bioconductor package for comprehensive visualization of genomic signal associations, BMC Genomics. 19 (2018) 234. doi:10.1186/s12864-018-4625-x.

Supplementary Figures

Figure S1

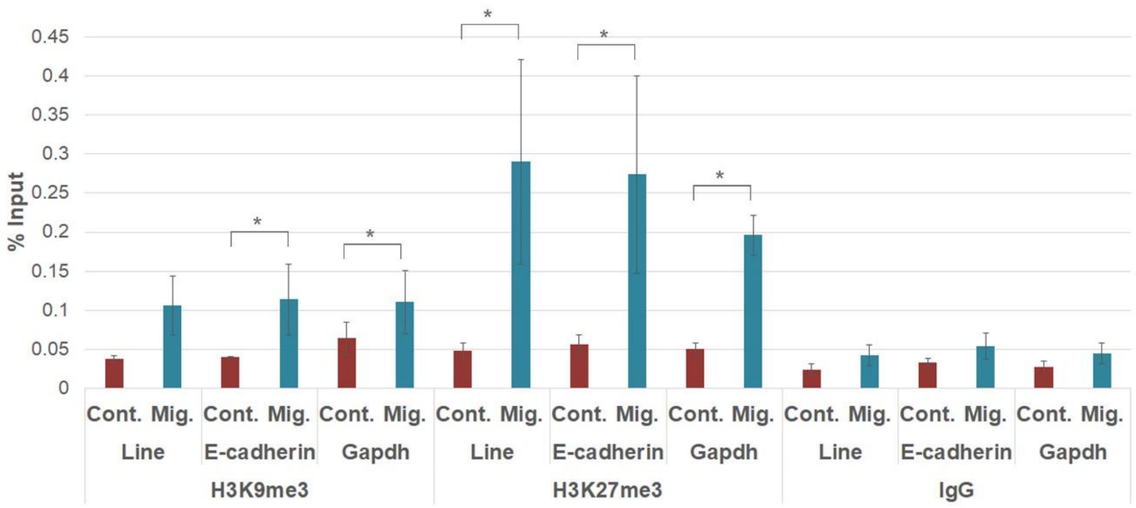

Figure S1: Increased levels of heterochromatin markers in the promoters of E-cadherin, Gapdh and Line upon induction of migration. ChIP-qPCR with antibodies against H3K9me3, H3K27me3 and control IgG. The immunoprecipitated DNA was analyzed with primers corresponding to the promoters of E-cadherin, Gapdh and Line in control cells (Cont.) and migrating cells (Mig.). Mean percentage of input ± SE, (n= 3) is represented in the graph. *P value<0.05 (Wilcoxon rank sum test).

Figure S2

a		# sequenced reads	# mapped reads	# unique mapped reads	coverage
H3K9me3	Cont.	$2.72*10^7$	99.96%	64.47%	0.96
	Mig.	$3.79*10^7$	99.96%	70.37%	1.34
H3K27me3	Cont.	$3.60*10^7$	99.96%	74.44%	1.28
	Mig.	$4.28*10^7$	99.97%	74.67%	1.52
H4K20me1	Cont.	$3.78*10^7$	99.80%	78.56%	0.75
	Mig.	$3.41*10^7$	99.92%	77.32%	0.68
Input	Cont.	$2.99*10^7$	99.98%	74.61%	1.06
	Mig.	$3.38*10^7$	99.98%	73.73%	1.2

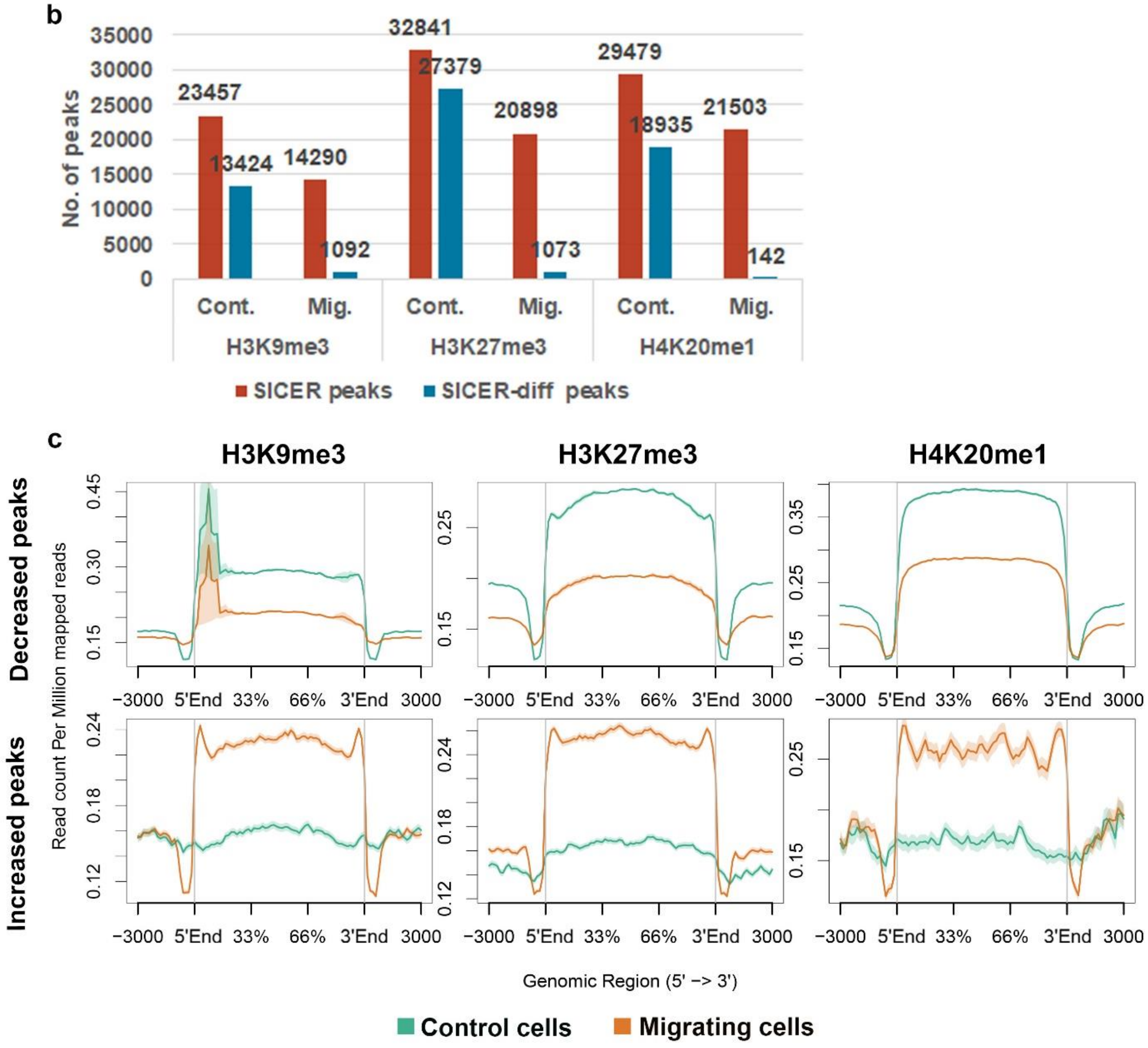

Figure S2: Enrichment of H3K9me3, H3K27me3 and H4K20me1 reads and peaks following ChIP-seq in control and migrating cells. (**a**) ChIP-seq statistics observed in control cells (Cont.) and migrating cells (Mig.). The percentage of reads mapped to the mouse genome (mm10) was calculated out of the total number of sequenced reads. Similarly, the percentage of reads mapped uniquely to the genome was calculated out of the total mapped reads. Coverage was calculated by dividing the number of sequenced nucleotides by the genome length. (**b**) The number of peaks and differential peaks (FDR<0.05) detected by SICER or SICER-diff, respectively, for the three modifications in control and migrating cells. (**c**) Distribution of H3K9me3, H3K27me3 and H4K20me1 signals in control cells (green line) and migrating cells (orange line) across differential peak regions. "Decreased peaks" (top) and “Increased peaks” (bottom) refer to regions where the signal was lower or higher, respectively, in migrating cells compared to control cells. In each graph, the region between 5’End and 3’End represents the analyzed peak body. Since peaks are of variable sizes, the coverage vectors were normalized to be equal length prior to average coverage calculation. The units for the abscissa are bp.

Figure S3

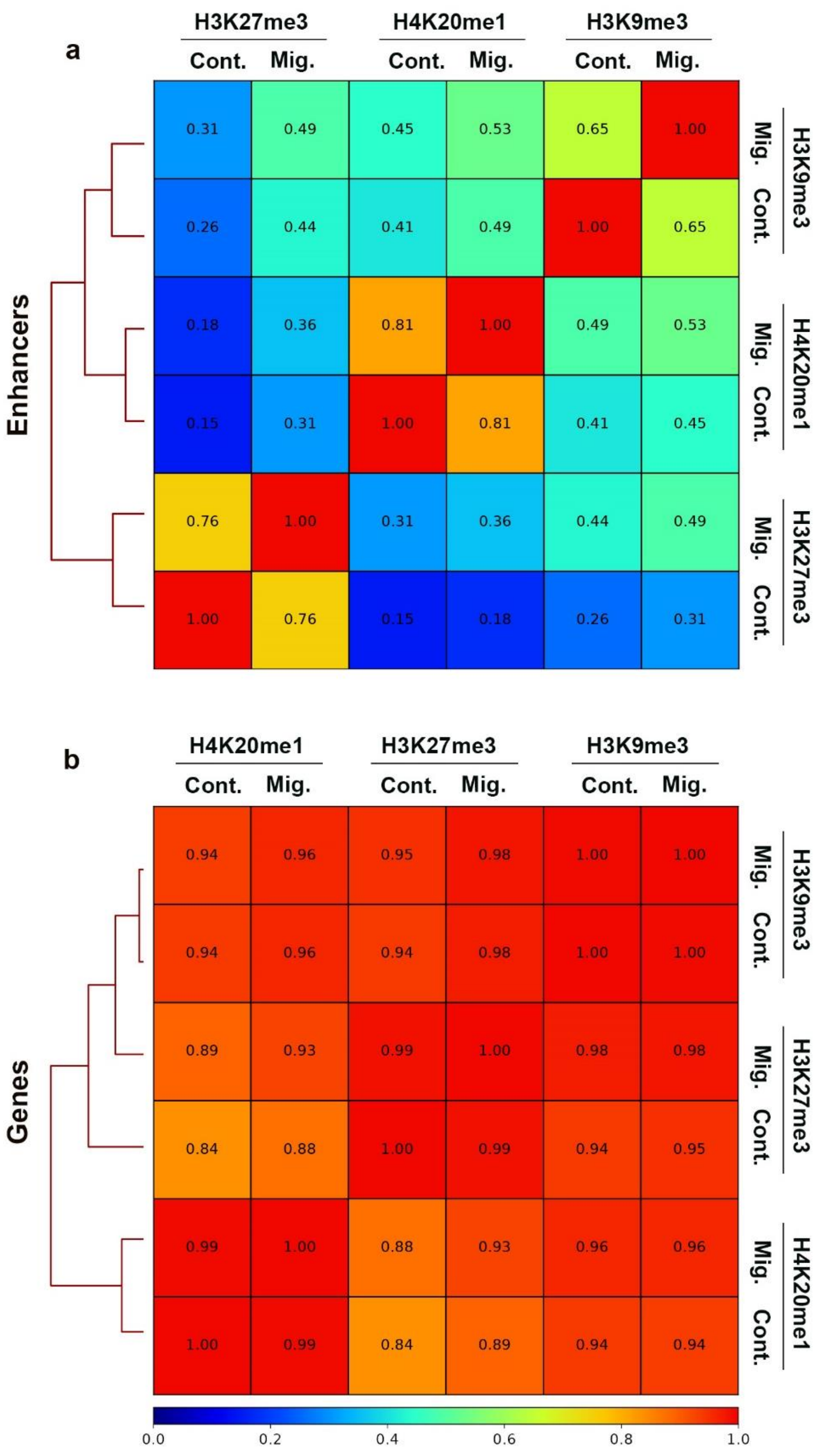

Figure S3: Correlation between H3K9me3, H3K27me2 and H4K20me1 ChIP-seq signal across specific genomic elements. Spearman correlation coefficients of the ChIP-seq signals

were calculated from reads coverage of consecutively equally sized 10kb bins for enhancers (a) and RefSeq genes (b).

Figure S4

		# sequenced reads	# mapped reads	# unique mapped reads
Cont.	Rep. 1	$3.80*10^7$	95.12%	94.06%
	Rep. 2	$5.50*10^7$	98.40%	92.91%
	Rep. 3	$3.75*10^7$	98.18%	92.99%
	Rep. 4	$2.66*10^7$	94.86%	93.09%
	Rep. 5	$2.35*10^7$	94.70%	93.10%
Mig.	Rep. 1	$3.08*10^7$	97.66%	94.00%
	Rep. 2	$4.93*10^7$	97.82%	92.84%
	Rep. 3	$3.25*10^7$	98.26%	92.72%
	Rep. 4	$2.81*10^7$	95.43%	92.80%
	Rep. 5	$3.01*10^7$	95.18%	92.81%
Mig. + GSK343	Rep 1	$2.32*10^7$	95.13%	92.57%
	Rep 2	$2.57*10^7$	95.40%	93.16%
	Rep 3	$2.65*10^7$	94.56%	92.57%
Cont. + DRB	Rep 1	$2.43*10^7$	98.53%	92.70%
	Rep 2	$2.41*10^7$	98.46%	92.07%
	Rep 3	$2.21*10^7$	98.45%	92.66%
Mig. + DRB	Rep 1	$2.21*10^7$	95.71%	92.02%
	Rep 2	$2.29*10^7$	98.57%	91.23%
	Rep 3	$2.47*10^7$	98.41%	91.64%

Figure S4: Transcriptome profiling of control and migrating cells using RNA-seq. RNA-seq statistics observed in control cells (Cont.), migrating cells (Mig.), GSK343-treated cells and DRB-treated cells. The percentage of mapped reads was calculated out of the number of sequenced reads. The percentage of unique mapped reads was calculated out of the total mapped reads.

Figure S5

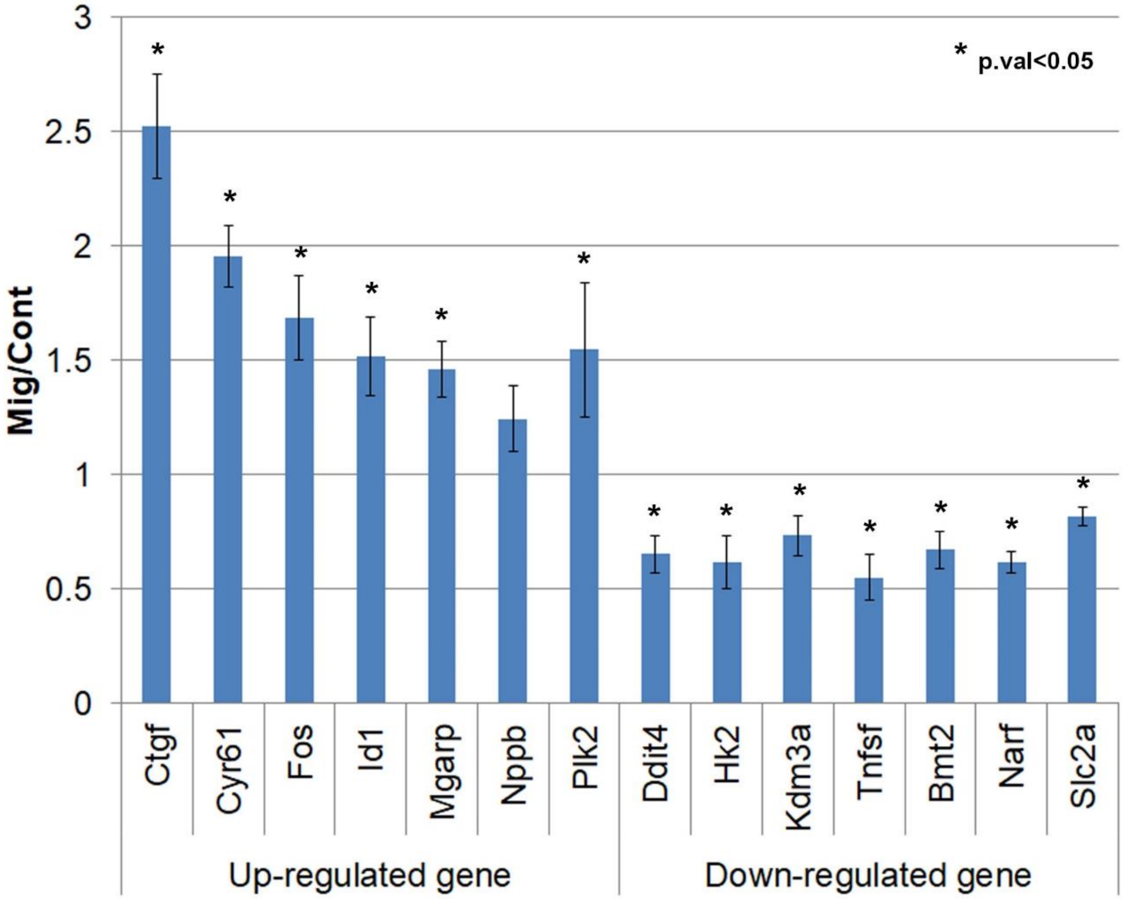

Sup.

Figure S5: Validation of RNA-seq based transcriptome profiling in control and migrating cells by RT-qPCR. Relative RNA levels of 7 migration up-regulated genes and 7 migration down regulated genes in the RNA-seq analysis were determined by RT-qPCR. The bars represent the average ratio between the RNA levels in migrating cells and the RNA levels in control cells in 3 repetitions ± SE. Statistical significance was calculated by student's T-test, * $p<0.05$.

Figure S6

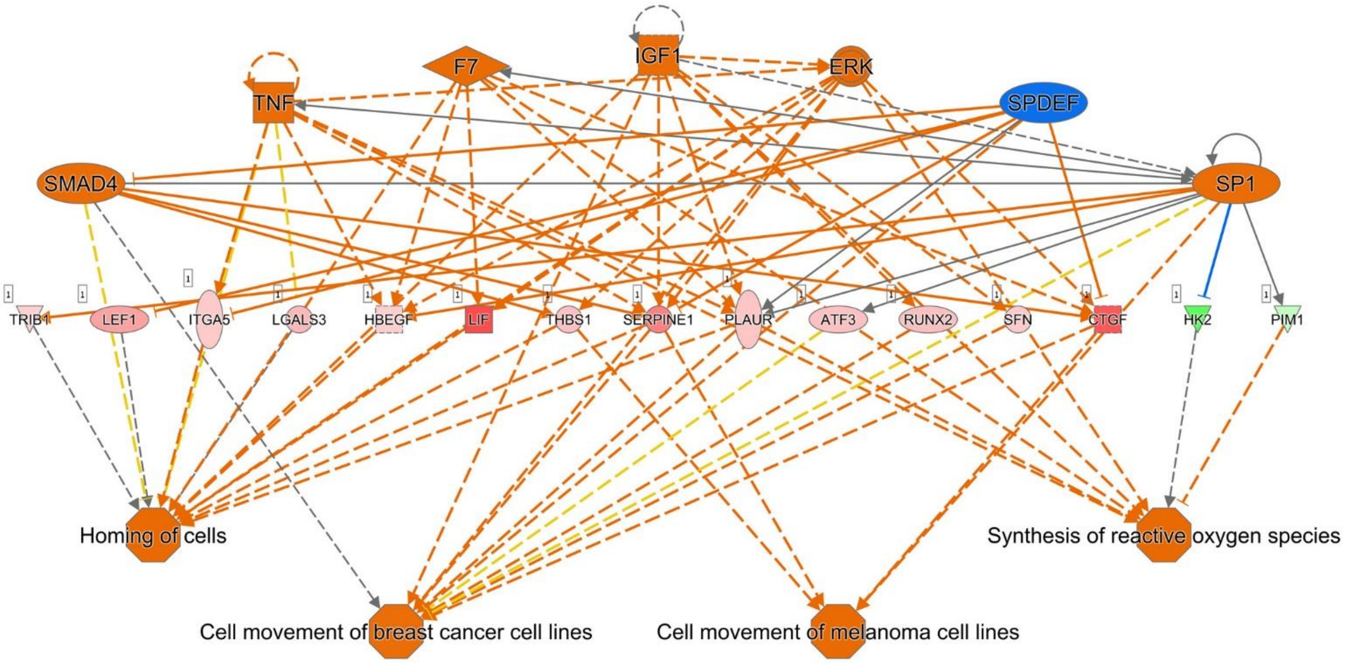

Figure S6: Predicted regulatory network effects identified from differential gene expression profile upon induction of migration. Upstream regulators predicted to be activated (orange) or inhibited (blue) appear in the upper layer. The presiction is based on the migration-induced differentially-expressed genes that appear in the middle layer (red represents upregulation and green represents downregulation). The shape of the nodes reflects the functional class of each gene product: transcription regulator (vertical ellipse), cytokine/growth factor (square), and complex/group/other (circle). In the lower layer, the expected phenotypic consequences of changes in gene expression are shown by considering the Ingenuity Knowledge Base (absolute z-score > 2 and P-value < 0.05). The orange octagonal symbols represent activation predicted relationships., Solid and dashed lines between genes represent known direct and indirect gene interactions, respectively. Orange (predicted to be activated) lines represent relationships with causal consistency, yellow for inconsistency, and gray for non-predicted effect.

Figure S7

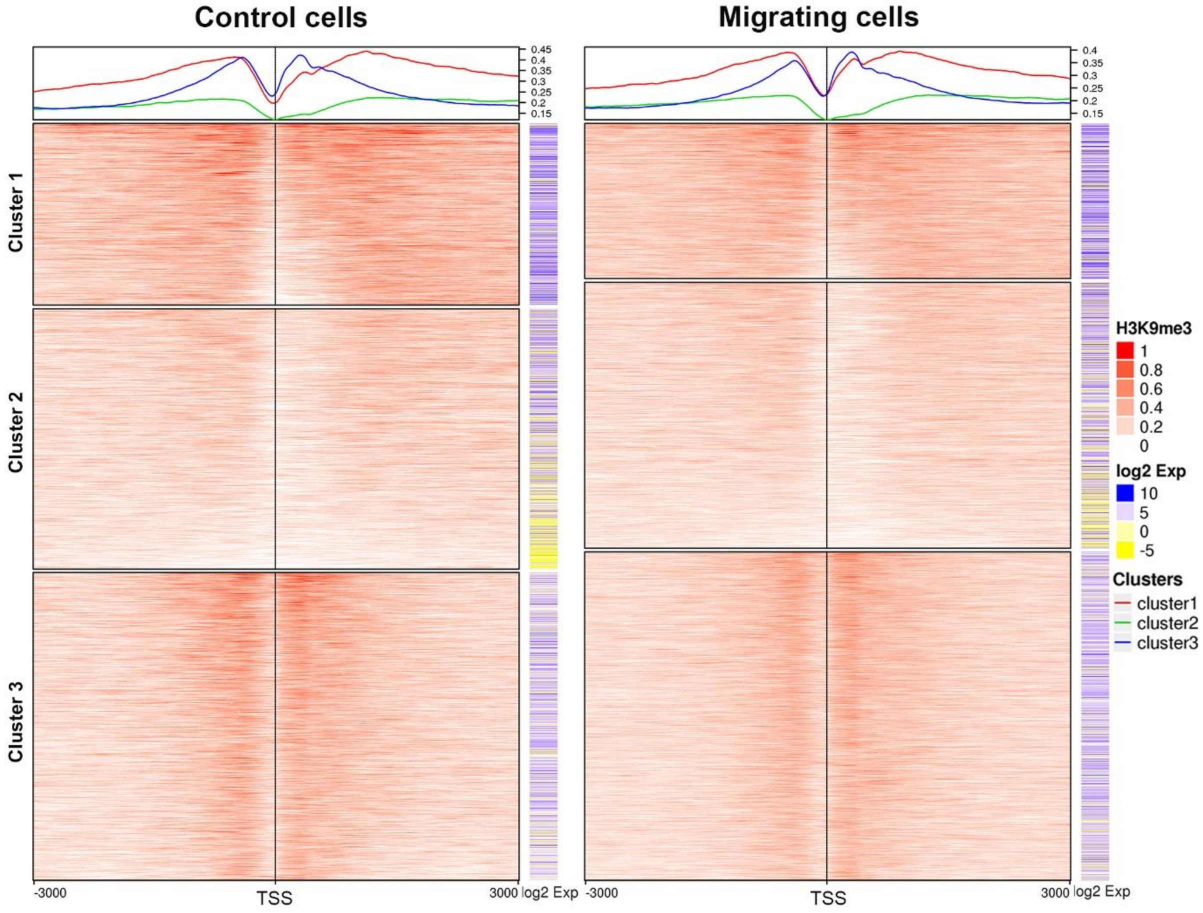

Figure S7: Association between H3K9me3 signal around the TSS and gene expression. ChIP-seq score of H3K9me3 around the TSS of H3K9me3-enriched genes ±3000bp was fetched and the average score across every 20 nucleotides was calculated and assembled into a matrix for creating the heatmap. The analysis was done for both control (left) and migrating cells (right). Each raw in the heatmap represents a gene (n= 13,226 in control cells, n= 10,554 in migrating cells). Genes were clustered according to their signal intensity using KNN clustering method (k=3). The expression level of every gene is presented next to its corresponding ChIP-seq signal line. The average ChIP-seq score distribution across genes belong to each cluster is presented above the heatmap.

Figure S8

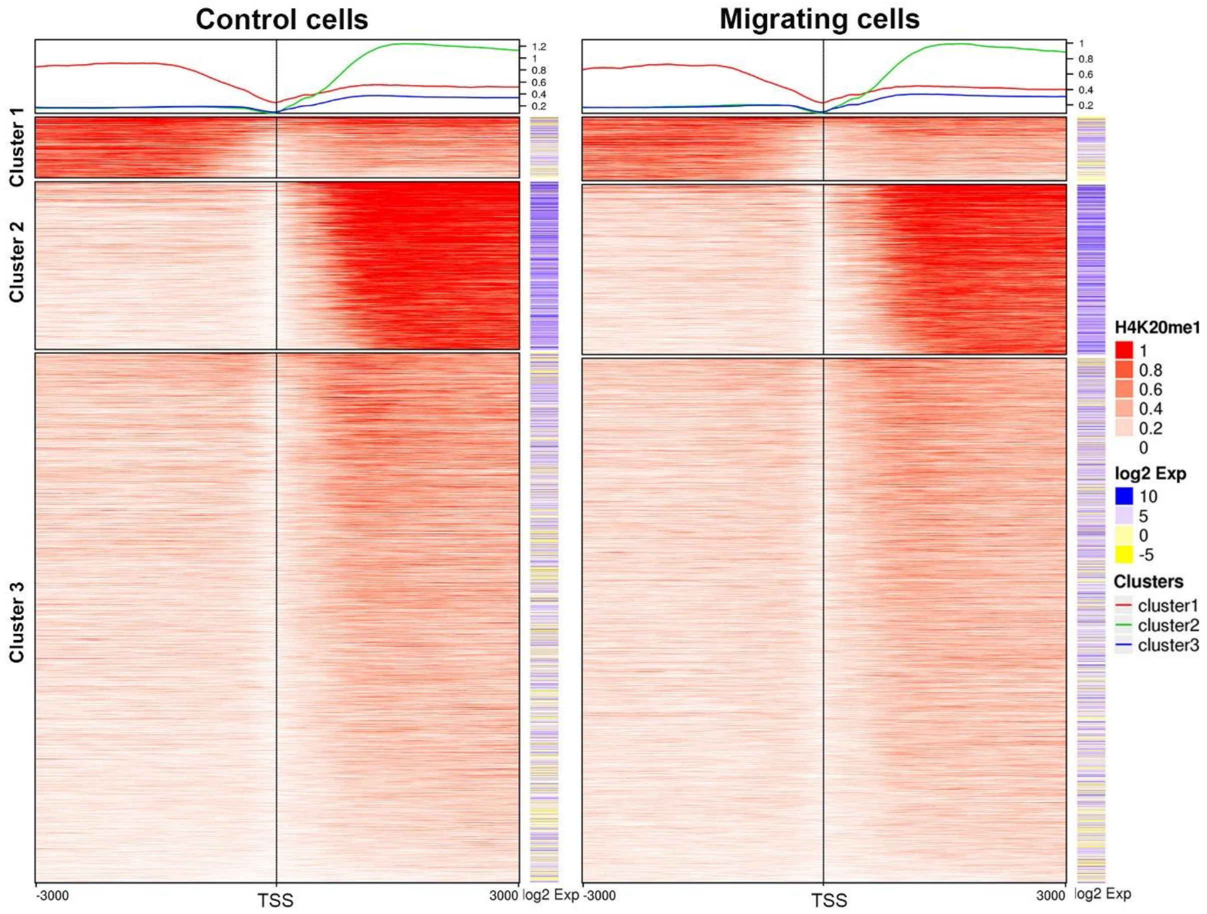

Figure S8: Association between H4K20me1 signal around the TSS and gene expression. ChIP-seq score of H4K20me1 around the TSS of H4K20me1-enriched genes ±3000bp was fetched and the average score across every 20 nucleotides was calculated and assembled into a matrix for creating the heatmap. The analysis was done for both control (left) and migrating cells (right). Each raw in the heatmap represents a gene (n= 18,056 in control cells, n= 15,865 in migrating cells). Genes were clustered according to their signal intensity using KNN clustering method (k=3). The expression level of every gene is presented next to its corresponding ChIP-seq signal line. The average ChIP-seq score distribution across genes belong to each cluster is presented above the heatmap.

Figure S9

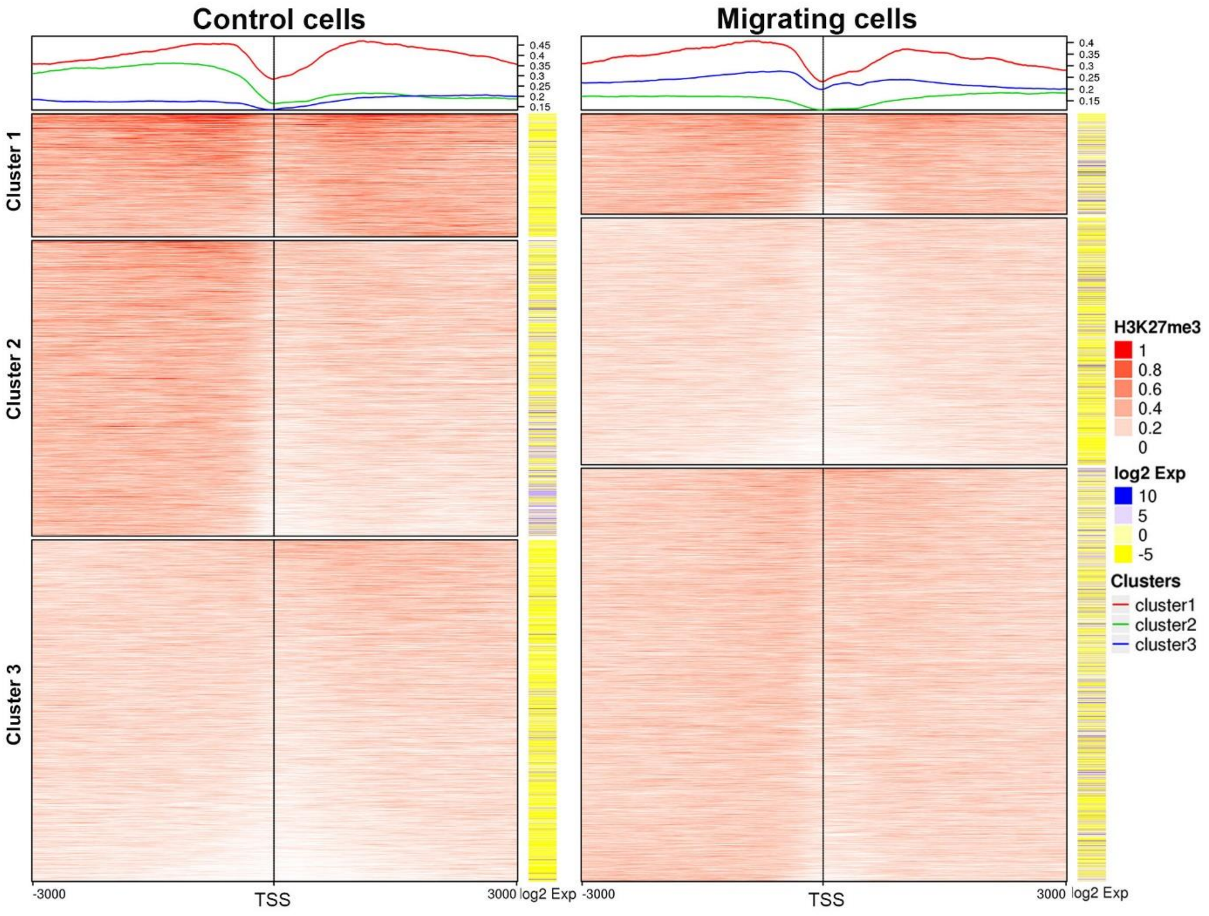

Figure S9: Association between H3K27me3 signal around the TSS and gene expression. ChIP-seq score of H3K27me3 around the TSS of H3K27me3-enriched genes ±3000bp was fetched and the average score across every 20 nucleotides was calculated and assembled into a matrix for creating the heatmap. The analysis was done for both control (left) and migrating cells (right). Each raw in the heatmap represents a gene (n= 9,713 in control cells, n= 7,633 in migrating cells). Genes were clustered according to their signal intensity using KNN clustering method (k=3). The expression level of every gene is presented next to its corresponding ChIP-seq signal line. The average ChIP-seq score distribution across genes belong to each cluster is presented above the heatmap.

Figure S10

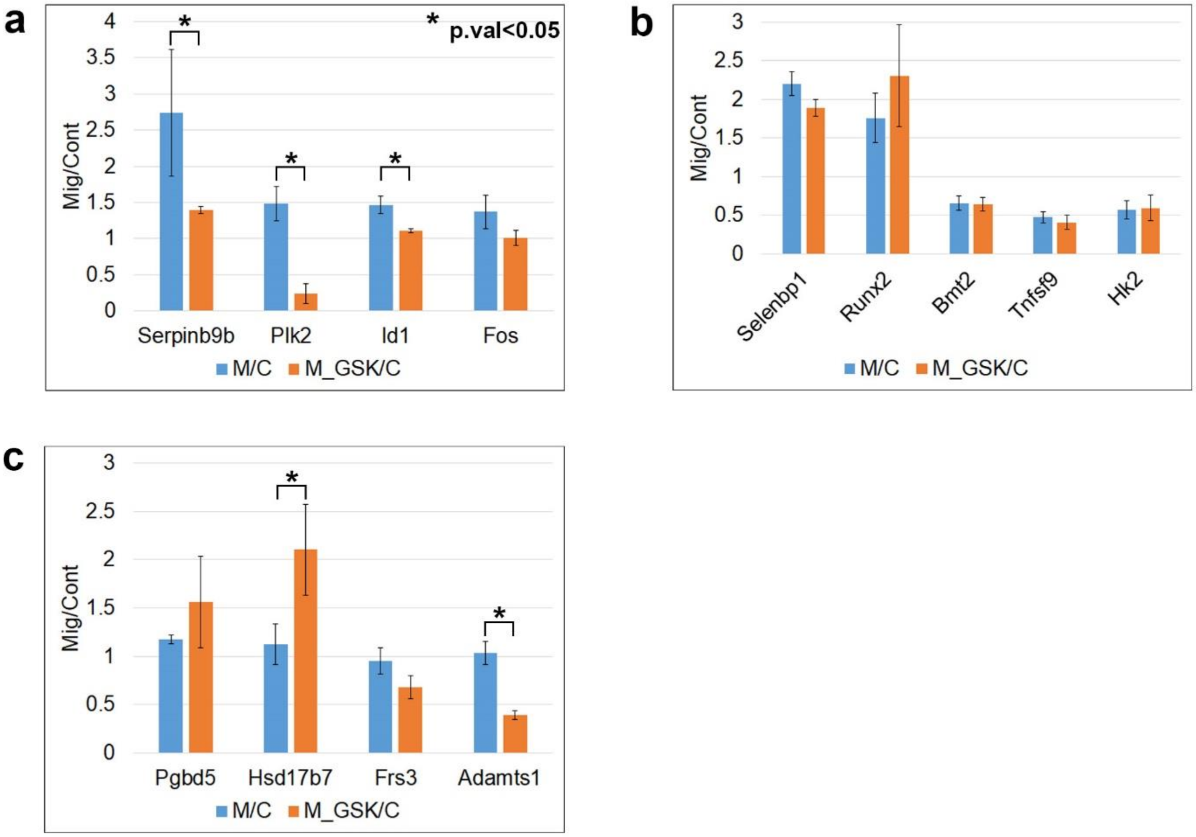

Figure S10: Validation of RNA-seq based transcriptome profiling in migrating cells treated with GSK343 by RT-qPCR. Relative RNA levels of K27me-dependent genes (a), K27me-independent genes (b) and K27me-buffered genes (c) in the RNA-seq analysis were determined by RT-qPCR. The blue bars and the orange bars represent the average ratio ± SE between the RNA levels in migrating cells and those in control cells or between the RNA levels in migrating cells treated with GSK343 and those in control cells, respectively. n=3, statistical significance was calculated by student’s T-test, * p<0.05.

Figure S11

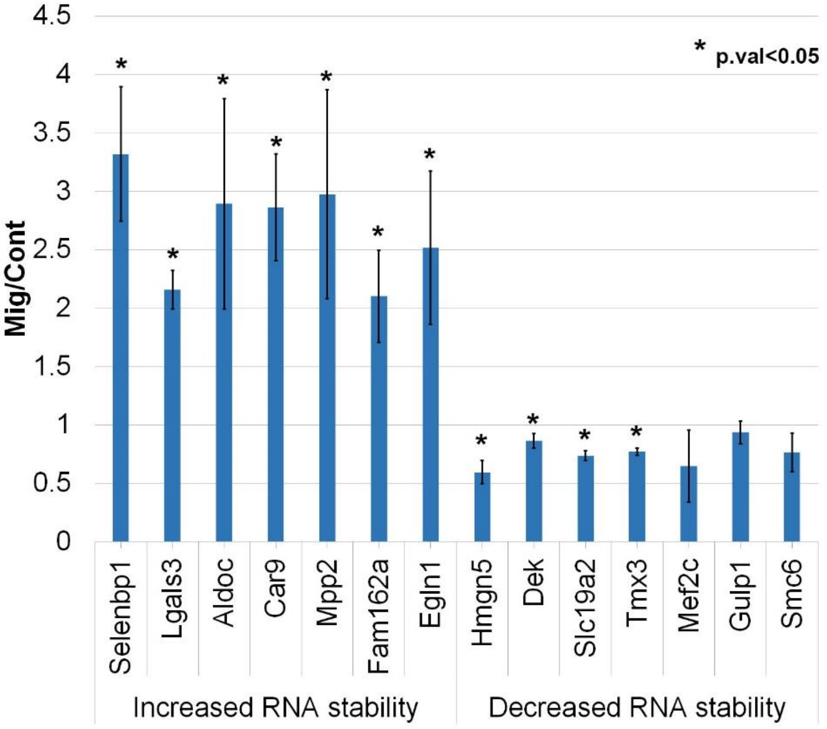

Figure S11: Validation of RNA-seq based RNA stability profiling in control and migrating cells by RT-qPCR. 7 genes with migration-increased RNA stability levels and 7 genes with migration-reduced RNA stability levels in the RNA-seq analysis were detected by RT-qPCR in control and migrating cells treated with DRB. The bars represent the average ratio between the RNA levels in migrating cells and the RNA levels in control cells in 3 repetitions ± SE. Statistical significance was calculated by student's T-test, * $p<0.05$.

Legends to Supplementary Tables

Sup. Table 1: Altered gene expression upon induction of migration. Differentially expressed genes between migrating cells and control cells with fold change >1.3 and FDR<0.05.

Sup. Table 2: The dependence of migration-induced transcriptional changes on H3K27 methylation. Differentially expressed genes between migrating cells and control that did not change between migrating cells treated with GSK343 and control cells were termed "H3K27me-dependent genes”. Differentially expressed genes between migrating cells and control cells and also between migrating cells treated with GSK343 and control cells were termed "H3K27me-independent genes”. Genes that were not changed between migrating cells and control cells but differentially expressed between migrating cells treated with GSK343 and control cells were termed "H3K27me-buffered genes". Genes with fold change >1.3 and a FDR<0.05 are listed.

Sup. Table 3: Migration induced changes in RNA stability. Differentially expressed genes between DRB-treated migrating cells and DRB-treated control cells (fold change >1.3 and FDR<0.05).

Supplementary Scripts

Script 1: Script to generate heatmaps presented in supplementary data.
